# Publisher Correction: DNA double-strand breaks in telophase lead to coalescence between segregated sister chromatid loci

**DOI:** 10.1038/s41467-019-11485-2

**Published:** 2019-08-02

**Authors:** Jessel Ayra-Plasencia, Félix Machín

**Affiliations:** 1 Unidad de Investigación, Hospital Universitario Nuestra Señora de Candelaria, Santa Cruz de Tenerife, Spain; 2 Escuela de Doctorado y Estudios de Posgrado, Universidad de La Laguna, Santa Cruz de Tenerife, Spain; 3 Instituto de Tecnologías Biomédicas, Universidad de La Laguna, Santa Cruz de Tenerife, Spain

**Keywords:** Checkpoints, Chromosome segregation, Mitotic spindle

Correction to: *Nature Communications* 10.1038/s41467-019-10742-8, published online 28 June 2019.

The original version of this Article contained an error in Fig. 6e, in which the labels ‘IAA’ and ‘Phle’ in the bottom right where inadvertently omitted. This has been corrected in both the PDF and HTML versions of the Article.

