# Supplementary material for: DNA double-strand breaks in telophase lead to coalescence between segregated sister chromatid loci
Source: Nat Commun. 2019 Jun 28;10:2862. doi: 10.1038/s41467-019-10742-8 (PMC6598993; doi:10.1038/s41467-019-10742-8)
Supplement: Supplementary file 1 — Supplementary Information [file 41467_2019_10742_MOESM1_ESM.pdf]

SUPPLEMENTARY INFORMATION

DNA double-strand breaks in telophase lead to coalescence between segregated sister chromatid loci.

Jessel Ayra-Plasencia[1,2], Félix Machín[1,3*].

[1] Unidad de Investigación, Hospital Universitario Nuestra Señora de Candelaria, Santa Cruz de Tenerife, Spain.

[2] Escuela de Doctorado y Estudios de Posgrado. Universidad de La Laguna, Santa Cruz de Tenerife, Spain

[3] Instituto de Tecnologías Biomédicas. Universidad de La Laguna, Santa Cruz de Tenerife, Spain.

* Contact: fmachin@funcanis.es

Unidad de Investigación, Hospital Universitario Nuestra Señora de Candelaria. Carretera del Rosario, 145. 38010. Santa Cruz de Tenerife, Spain.

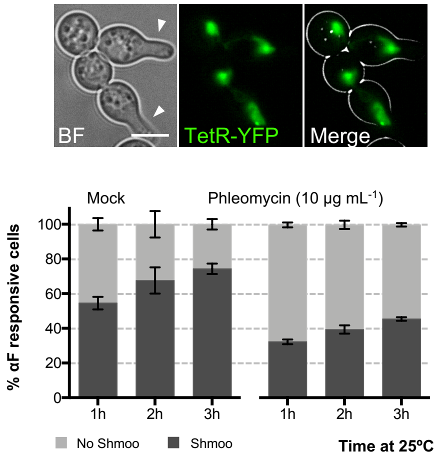

Supplementary Figure 1. Responsiveness to αF (as a marker of G_1) is delayed after DNA damage in telophase. FM593 was treated as described in Figure 1b. On the top, a representative micrograph showing two telophase-like cells (binucleated dumbbells) in which one daughter cell responds to αF by acquiring the shmoo morphology (filled arrowheads). Scale white bar corresponds to 5 µm; BF, bright field. At the bottom, chart depicting the evolution of the αF-responsive cells after the telophase release (mean ± s.e.m., n=3 independent experiments). Source data are provided as a Source Data file.

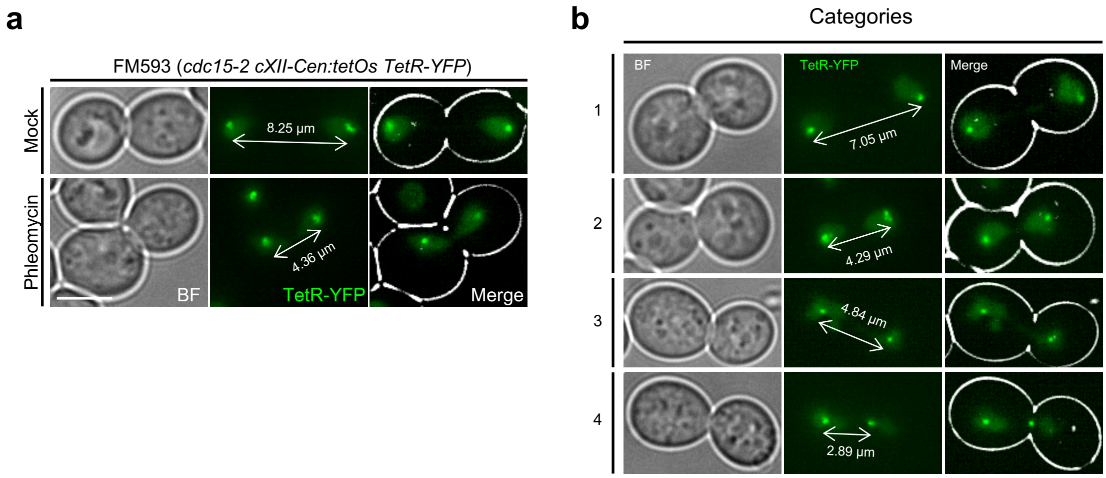

Supplementary Figure 2. (a) Representative cells from Figure 2a (1h after initiation of treatment). Note how sister centromeres are kept close to opposite poles throughout the telophase block. DNA damage after phleomycin addition breaks this disposition so the inter sister centromere distance is reduced. (**b**) Examples of cell categories included in the Figure 2b. Note how in category 4 one of the sister centromeres is at the bud neck. Scale bar corresponds to 5 µm; BF, bright field.

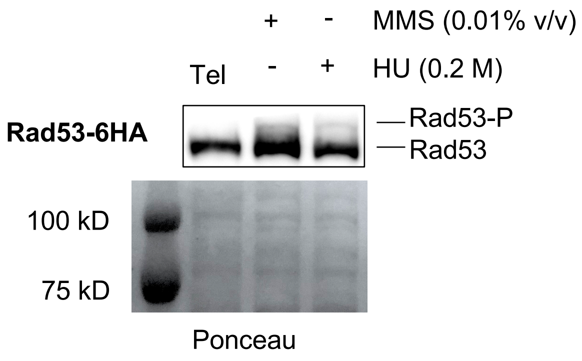

Supplementary Figure 3. DNA damages other than DSBs do not trigger a strong DNA Damage Response in telophase. Related to Figure 2c. Rad53 is weakly phosphorylated in telophase after treatments with the DNA alkylating agent MMS or the ribonucleotide reductase inhibitor hydroxyurea (HU). Strain FM2329 was arrested for 3h at telophase before splitting the culture in two. MMS and HU were added to the indicated subcultures and cells were harvested after 1h for Western blot analysis. Tel, sample at the telophase arrest, before splitting the culture in two. The leftmost lane in the Ponceau staining corresponds to the protein weight markers (shared with the Western blot shown in Supplementary Figure 7c). Source data are provided as a Source Data file.

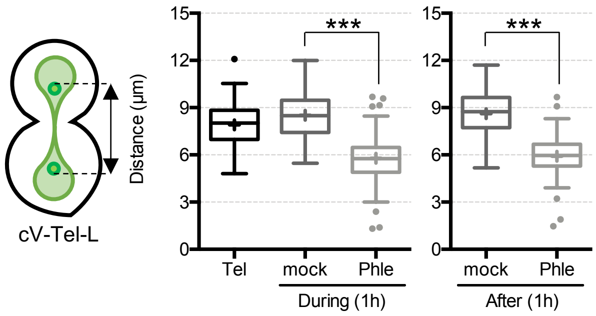

Supplementary Figure 4. Telomeres other than cXIIr-Tel get closer after DSBs in telophase. Related to Figures 2 and 3. Strain FM567 was treated as in Figure 2a and samples taken at the indicated experimental time points for fluorescence microscopy. The distance between sister chromosome V left telomeres was measured and box-plotted (*** indicates p<0.0001 in mock/phleomycin comparisons at each time point; Mann-Whitney U Test). Source data are provided as a Source Data file.

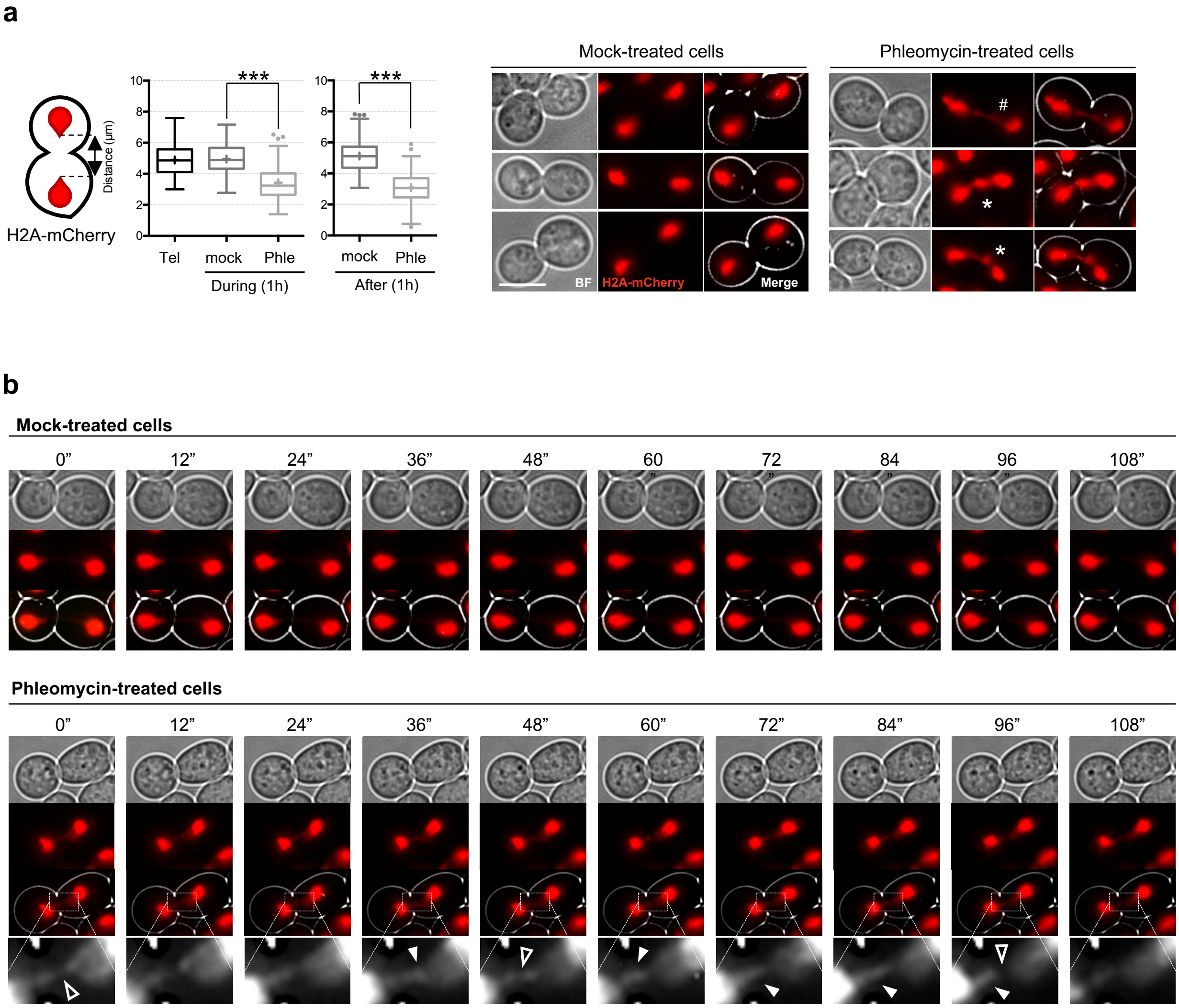

Supplementary Figure 5. The bulk of segregated nuclear masses moves closer and forms *de novo* chromatin bridges after DNA damage in telophase. FM2354 was treated like in Figure 2a. **(a)** Boxplots of minimum distances between bright (condensed) histone-labelled segregated nuclear masses (*** indicates p<0.0001; Mann-Whitney U Test). On the right, representative cells observed during treatments. Note the presence of distinct chromatin bridges in phleomycin formed by either thin histone-poor inter nuclear connections (#) or bulgy string-like bridges (*). For the plot, it was considered for calculation of the minimum distances neither the bulgy minor chromatin fraction in the bridge nor the histone-poor signal along the bridge. (**b**) Time frames of representative cell examples during mock (above) and phleomycin (below) treatments. Phleomycin led to the two above-mentioned outcomes. Here it is represented the dynamics of a *de novo* bridge comprised of apparently decondensed and histone-poor DNA (zoomed in at the bottom). Filled arrowheads point to histone-poor DNA moving along the bridge, whereas hollow arrowheads show empty spaces. For the second outcome (bulgy string-like bridge) refer to Figure 3e. BF, bright field; scale white bar represents 5 µm. Source data are provided as a Source Data file.

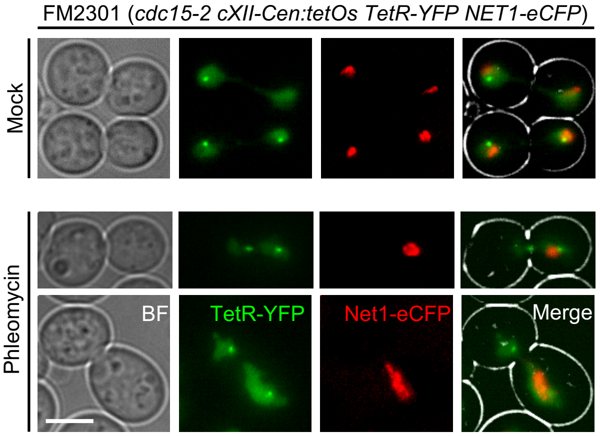

Supplementary Figure 6. Representative cells from Figure 2f (1h after initiation of treatment). Note how sister rDNAs (coated with Net1-eCFP) merged into a single nucleolus upon phleomycin treatment. BF, bright field; scale white bar represents 5 µm.

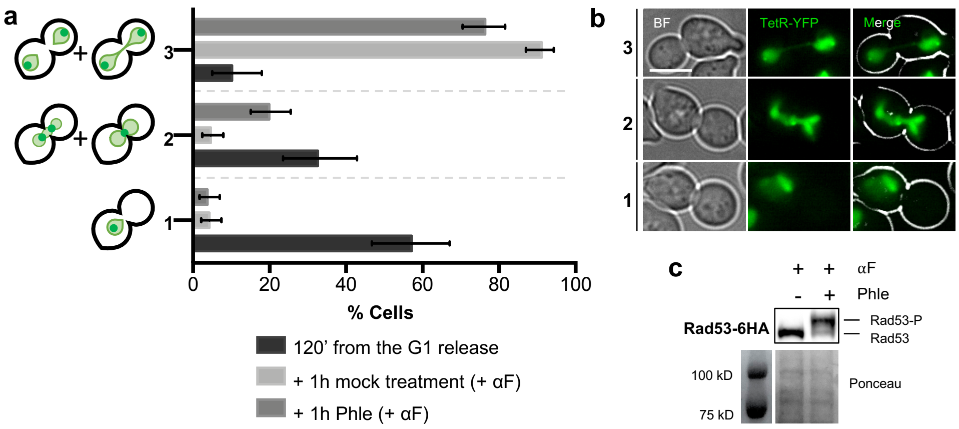

Supplementary Figure 7. Sister loci stay close more often when DSBs are generated in cells normally transiting through anaphase. Strain FM593 was release from a G1 arrest at 25 ºC and monitor each 10' seeking the beginning of chromosome segregation. When cells were entering anaphase (120' from G_1 release in this particular experiment), the culture was split in two and phleomycin was added to one subculture. To simplify subsequent cell figures, αF was also added to both subcultures. (**a**) Categorization of cell and nuclear morphologies (soluble tetR-YFP pool) at the indicated experimental points (± CI95). (**b**) Representative cells for each category in the subculture treated with phleomycin. (**c**) Western blot for Rad53 showing that phleomycin elicited a strong DNA damage response. Note that when phleomycin was added cells underwent progression into anaphase (i.e., category 1 of mononucleated dumbbells accounts for only 5% of cells at the end point); however, up to 20% of cells have an elongated nucleus with two very close centromeres. Rad53 was highly hyperphosphorylated, pointing out that a strong DNA damage response also occurs if DSBs are generated in anaphase. The leftmost lane in the Ponceau staining corresponds to the protein weight markers (shared with the Western blot shown in Supplementary Figure 3). Source data are provided as a Source Data file.

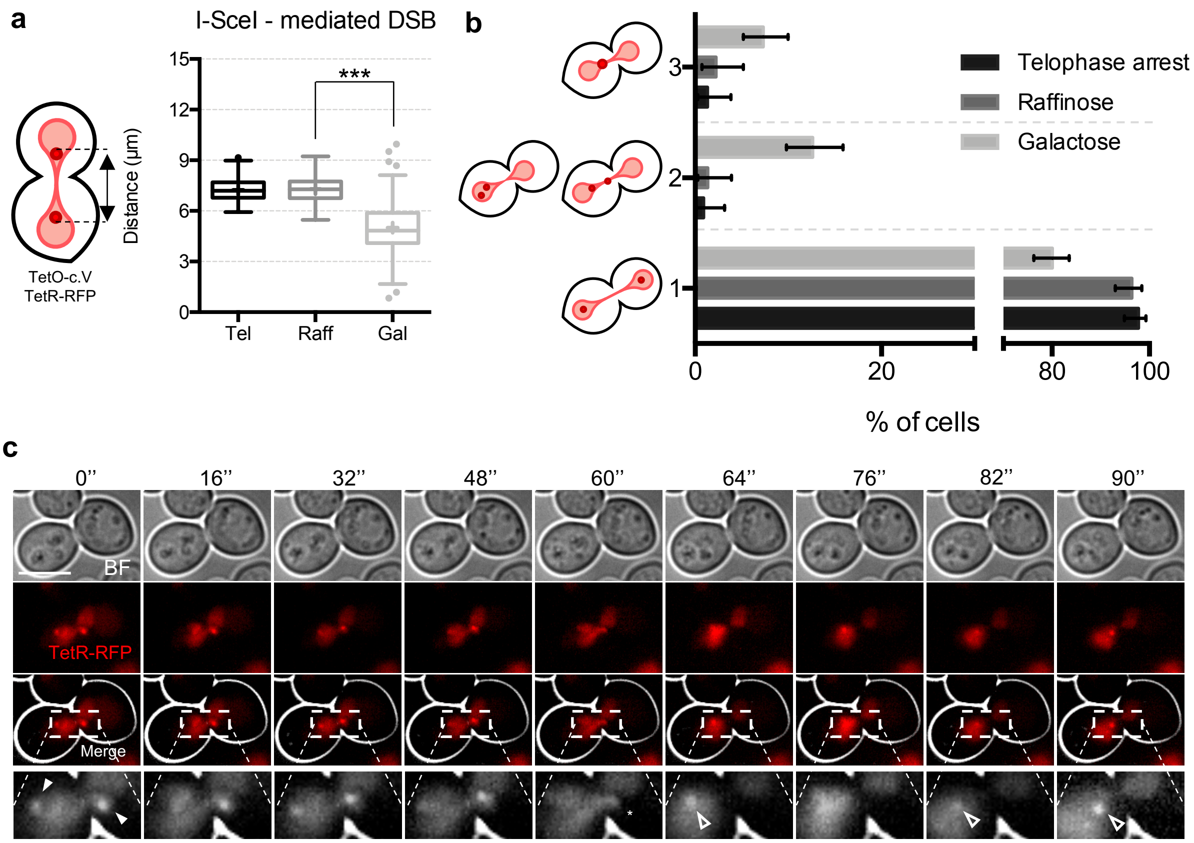

Supplementary Figure 8. DSBs generated by endonucleases also lead to approximation and coalescence of adjacent sister loci. The strain FM2456 was grown at 25 ºC in raffinose (2% w/v) and arrested in telophase (3h at 37 ºC) before splitting the culture in two (Tel). One subculture was left in raffinose for another hour (Raff), while galactose (2% w/v) was added to the second one to induce the I-SceI endonuclease (Gal). Samples were taken at the indicated experimental points for analysis under the fluorescence microscope. (**a**) the distance between the sister loci adjacent to the I-SceI recognition site was measured and box-plotted (*** indicates p<0.0001; Mann-Whitney U Test). Note that cells with only one focus (coalescent sister loci) were omitted in the box plots. (**b**) Categorization of sister loci as in Figure 3a (± CI95). (**c**) A sample of "Gal" was filmed for 2 minutes. A representative cell with dynamic coalescence near the bud neck is shown. Source data are provided as a Source Data file.

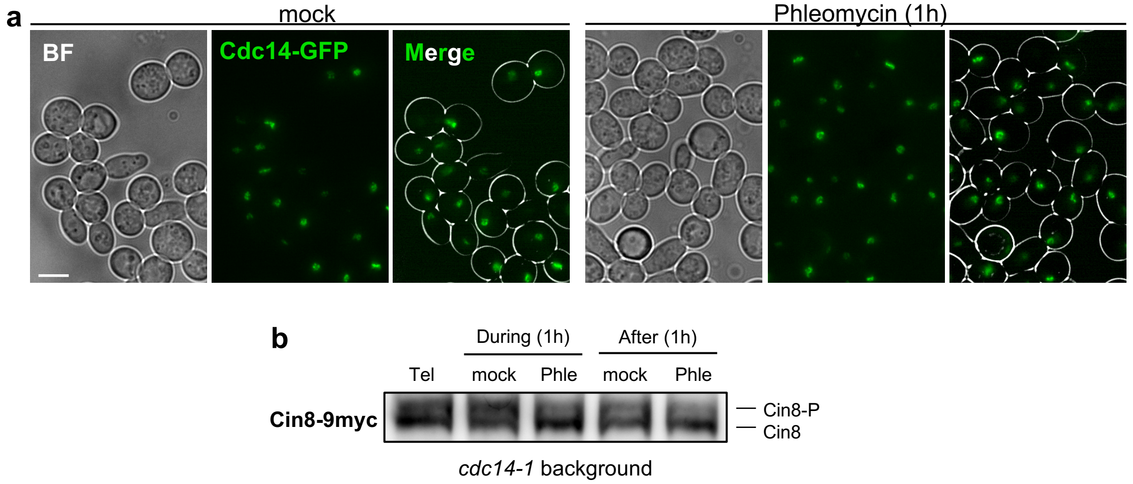

Supplementary Figure 9. Cdc14 does not dephosphorylate Cin8 upon DSB generation in telophase. (a) Cdc14 remains in the nucleolus upon phleomycin treatment in telophase. The strain FM2302 was treated as in Figure 2a. One hour within phleomycin (or mock) incubation, samples were photographed under the microscope. Note that Cdc14-GFP appears as concentrated clusters, lines or small loops; all characteristics of rDNA in telophase. **(b)** The strain FM2478 was treated and samples processed as in Figure 6b. BF, bright field. Source data are provided as a Source Data file.

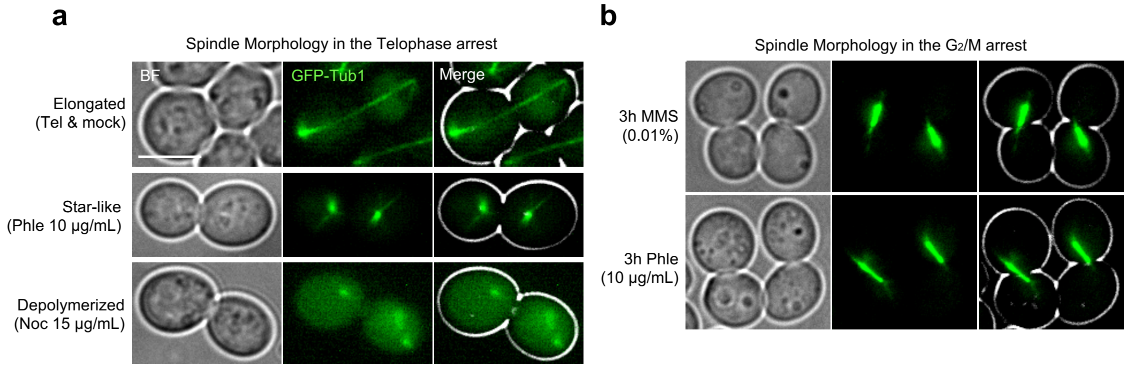

Supplementary Figure 10. Phleomycin does not depolymerize microtubules. (a) Representative Tub1 figures after telophase cells are (i) left untreated, (ii) treated with phleomycin or (iii) treated with nocodazole. The elongated spindle was the major phenotype at the telophase block (3h) or after 1h of mock treatment. Phleomycin yielded the star-like morphology with or without evident interpolar microtubules (iMTs). Here a representative cell without iMTs is shown in order to compare with the extreme Noc phenotype. For cells with iMTs see Figure 5a. For iMTs changing dynamics see Supplementary Movies 11-14. Unlike phleomycin, Nocodazole caused Tub1 to appear soluble throughout the cell, with no signs of organized MTs and only a Tub1 fraction concentrated as one focus per cell body (probably at the SPBs). (**b**) DNA damage exerted with Phleomycin over an asynchronous culture induced a G_2/M block (mononucleated dumbbell cell) with a characteristic short and thick spindle, similar to the G_2/M blocks caused by other DNA damaging agents (MMS in included for comparison). BF, bright field; scale white bar represents 5 µm.

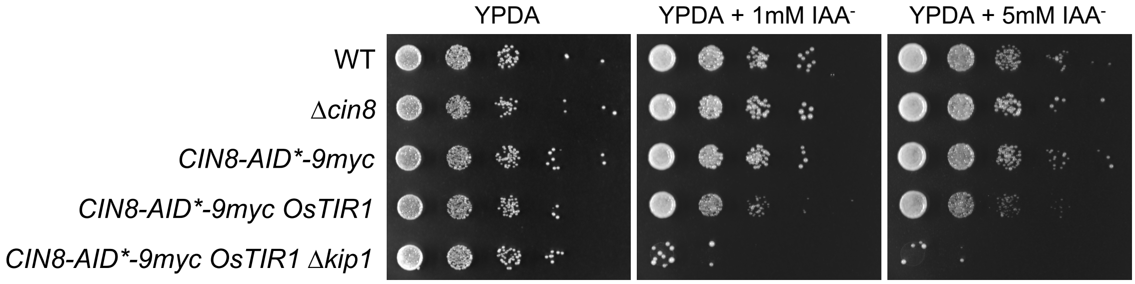

Supplementary Figure 11. Cin8-aid levels drop beyond functionality after auxin addition. Strains FM518, FM2461, FM2465, FM2466 and FM2473 were grown in YPD, normalized to 0.5 OD_{600}, 1:10 serially diluted and spotted onto the indicated plates. Note that loss of Cin8 is not deleterious unless it is combined with a *kip1*Δ mutant. We made use of this synthetic lethality to confirm auxin-mediated degradation of Cin8-aid.

Supplementary Table 1. Strains used in this work.

Strain	Genotype	Origin
AS499 (YPH499)	*MATa ura3-52 lys2-801 ade2-101 trp1-Δ63 his3-Δ200 leu2-Δ1 bar1-Δ*	A. Strunnikov [a]
FM593	AS499; *ade2-101:TetR-YFP:ADE2; cXIIr(194Kb):tetOs:HIS3; cdc15-2:9myc:Hph*	F. Machín [b]
FM588	AS499; *ade2-101:TetR-YFP:ADE2; cXIIr(1061Kb):tetOs:HIS3; cdc15-2:9myc:Hph*	F. Machín [b]
FM2329	FM593; *RAD53:6HA:KanMX4*	This Study
FM2323	FM593; *SIC1:6HA:KanMX4*	This Study
FM2329	FM593; *HTA2:mCherry:KanMX4*	This Study
FM2301	FM593; *NET1:eCFP:KanMX4*	This Study
FM2381	AS499; *ura3-52:GFP:TUB1:URA3; cdc15-2:9myc:Hph*	This Study
FM2316	FM593; *SPC42:mCherry:KanMX6*	This Study
FM2317	FM593; *CIN8:mCherry:KanMX6*	This Study
FM2335	FM593; *CIN8:9myc:natNT2*	This Study
FM916	FM588; Δ*rad9::natMX*	This Study
FM2477	FM916; *SIC1:6HA:KanMX4*	This Study
FM567	*MATa trp1-1 his3-11,15 leu2-3,112*; *Telomere cV-L:tetO:LEU2; ade2-1:URAp-TetR-YFP:ADE2; Telomere cV-R:lacO:TRP1; ura3-1:HISp-CFP-lacI:URA3*; *cdc15-2:9myc:Hph*	F. Machín [b,c]
FM2448 (W303)	*MATα leu2-3,112 trp1-1 can1-100 ura3-1 ade2-1 his3-11,15 RAD5*; *cdc15-2*	This study
LSY3902	W303*; MATa lys2::GAL-I-SceI Ura3:tetO Leu2-LacO(YEL023C) HIS3-YFP-LacI TetR-RFP RAD52-CFP*	L.S. Symington [d]
FM2456*	W303*; MATa lys2::GAL-I-SceI Ura3-tetO Leu2-LacO(YEL023C) HIS3-YFP-LacI TetR-RFP; cdc15-2*	This study [c]
FM889	FM588; Δ*rad52::KanMX*	F. Machín [e]
LGY2055	W303*; MATa* Δ*cin8::ura3MX; leu2-3,112::CIN8-3GFP-LEU2*	Gift from Larisa Gheber
LGY2058	W303*; MATa* Δ*cin8::ura3MX; leu2-3,112::CIN8-3A-3GFP-LEU2*	Gift from Larisa Gheber

LGY2575	W303; *MATa Δcin8::ura3MX; leu2-3,112::CIN8-3D-3GFP-LEU2*	Gift from Larisa Gheber
FM2505*	W303; *MATα Δcin8::ura3MX; leu2-3,112::CIN8-3GFP-LEU2; cdc15-2*	This Study
FM2506*	W303; *MATα Δcin8::ura3MX; leu2-3,112::CIN8-3A-3GFP-LEU2; cdc15-2*	This Study
FM2507*	W303; *MATa Δcin8::ura3MX; leu2-3,112::CIN8-3D-3GFP-LEU2; cdc15-2*	This Study
FM1293	AS499; *cdc15-2:9myc:Hph*	This Study
FM2461	FM1293; *Δcin8::HIS3MX4*	This Study
FM2465	FM593; *CIN8:AID*:9myc:KanMX*	This Study
FM2466	FM2465; *ura3-52::ADH1-OsTIR1-9myc-URA3*	This Study
FM2473	FM2466; *Δkip1::HIS3MX4*	This Study
FM2302	AS499; *CDC14:GFP:KanMX; cdc15-2:9myc:Hph*	This Study
FM518	AS499; *ade2-101:TetR-YFP:ADE2; cXIIr(487Kb):tetOs:HIS3; cdc14-1:9myc:TRP1*	F. Machín [b]
FM2478	FM518; *CIN8:9myc:KanMX4*	This Study

[a] Parental strain; a *bar1-Δ* a derivative of YPH499, congenic to S288C.

[b] Quevedo, O., García-Luis, J., Matos-Perdomo, E., Aragón, L., and Machín, F. (2012). Nondisjunction of a single chromosome leads to breakage and activation of DNA damage checkpoint in g2. PLoS Genet. *8*, e1002509.

[c] These strains carry either lacI-YFP or lacI-CFP as fluorescent reporters, along with TetR-RFP or TetR-YFP. We were unable to detect *lacO*-based foci at 34-37 ºC (used for the telophase arrest in *cdc15-2* strains).

[d] Oh, J., Lee, S.J., Rothstein, R., and Symington, L.S. (2018). Xrs2 and Tel1 Independently Contribute to MR-Mediated DNA Tethering and Replisome Stability. Cell Reports. 25(7):1681-1692.e4.

[e] García-Luis, J., and Machín, F. (2014). Mus81-Mms4 and Yen1 resolve a novel anaphase bridge formed by noncanonical Holliday junctions. Nature communications. 5:5652.

[*] Strains created by crosses with FM2448, followed by spore selection for the annotated genotypes.
