# Supplementary material for: DNA double-strand breaks in telophase lead to coalescence between segregated sister chromatid loci
Source: Nat Commun. 2019 Jun 28;10:2862. doi: 10.1038/s41467-019-10742-8 (PMC6598993; doi:10.1038/s41467-019-10742-8)
Supplement: Supplementary file 3 — Description of Additional Supplementary Files [file 41467_2019_10742_MOESM3_ESM.pdf]

Description of Additional Supplementary Files

File Name: Supplementary Movie 1
Description: Videomicroscopy (2 min, 2 sec per frame) of a cell showing stable coalescence of sister cXIIr telomeres. Note that when both sister telomeres merge they start moving around together.

File Name: Supplementary Movie 2
Description: Videomicroscopy (2 min, 2 sec per frame) of a cell showing stable coalescence of sister cXIIr telomeres. Note that both sister telomeres are together most of the time but transiently disengaged for 2-4 seconds.

File Name: Supplementary Movie 3
Description: Videomicroscopy (2 min, 2 sec per frame) of a cell showing unstable coalescence of sister cXIIr telomeres. Note how both sister telomeres get together and then quickly split apart.

File Name: Supplementary Movie 4
Description: Videomicroscopy (2 min, 2 sec per frame) of the representative phenotype for the bulk of the segregated chromatin during the mock-treatment in telophase. Note how the histone-labelled DNA masses are far apart, homogenously condensed and without a bridge connecting both masses.

File Name: Supplementary Movie 5
Description: Videomicroscopy (2 min, 2 sec per frame) of a representative cell for one of the major phenotypes observed 1 h after phleomycin addition: a histone-poor (decondensed?) dynamic bridge that connects the bulk of the segregated chromatin. Histone-labelled DNA seems to travel along the bridge. This is example 1 of this phenotype.

File Name: Supplementary Movie 6
Description: Videomicroscopy (2 min, 2 sec per frame) of a representative cell for one of the major phenotypes observed 1 h after phleomycin addition: a histone-poor (decondensed?) dynamic bridge that connects the bulk of the segregated chromatin. Histone-labelled DNA seems to travel along the bridge. This is example 2 of this phenotype.

File Name: Supplementary Movie 7
Description: Videomicroscopy (2 min, 2 sec per frame) of a representative cell for the other major phenotypes observed 1 h after phleomycin addition: a bridge comprised of bulgy domains. In this case, the origin of the bulge is shown. The bulge arises when part of the chromatin pops up from one of the segregated masses.

File Name: Supplementary Movie 8
Description: Videomicroscopy (2 min, 2 sec per frame) of a cell showing unstable coalescence of sister loci adjacent to DSB(s) generated endonucleolytically (I-SceI). Note how one locus stays near the bud neck for a while before seeking its sister into the corresponding cell body. Note also that just when passing through the neck the locus is briefly seen as an elongated linear signal rather than a focus.

File Name: Supplementary Movie 9
Description: Videomicroscopy (2 min, 2 sec per frame) of a cell responding to DSB(s) generated endonucleolytically (I-SceI). In this case, note how the locus that goes through the bud neck is again seen as an elongated linear signal (or briefly as two foci) rather than a focus.

File Name: Supplementary Movie 10
Description: Videomicroscopy (2 min, 2 sec per frame) of a third example of cells responding to DSB(s) generated endonucleolytically (I-SceI). In this case, note how the locus passing through the bud neck was trapped in the elongated linear form for a few seconds before merging with its sister in one of the daughter nuclei.

File Name: Supplementary Movie 11
Description: Videomicroscopy (2 min, 2 sec per frame) of a cell showing one class of microtubule morphology after 1 h in phleomycin: shorter but stable spindle.

File Name: Supplementary Movie 12
Description: Videomicroscopy (2 min, 2 sec per frame) of a cell showing a second class of microtubule morphology after 1 h in phleomycin: spindle with apparently detached interpolar MTs.

File Name: Supplementary Movie 13
Description: Videomicroscopy (2 min, 2 sec per frame) of a cell showing how detached interpolar MTs get eventually attached again. Also note how one MT center (i.e., SPB) transiently crosses the bud neck and is then quickly pulled back to the daughter cell.

File Name: Supplementary Movie 14
Description: Videomicroscopy (2 min, 2 sec per frame) of a cell showing a third class of microtubule morphology after 1 h in phleomycin: no apparent iMTs (G1-like morphology). Also note how one MT center (i.e., SPB) transiently crosses the bud neck and is then quickly pulled back to the daughter cell.
