# Supplementary material for: DNA double-strand breaks in telophase lead to coalescence between segregated sister chromatid loci
Source: Nat Commun. 2019 Jun 28;10:2862. doi: 10.1038/s41467-019-10742-8 (PMC6598993; doi:10.1038/s41467-019-10742-8)
Supplement: Supplementary file 18 — Reporting Summary [file 41467_2019_10742_MOESM18_ESM.pdf]

nature research

Corresponding author(s): Félix Machín

Reporting Summary

Nature Research wishes to improve the reproducibility of the work that we publish. This form provides structure for consistency and transparency in reporting. For further information on Nature Research policies, see Authors & Referees and the Editorial Policy Checklist.

Statistical parameters

When statistical analyses are reported, confirm that the following items are present in the relevant location (e.g. figure legend, table legend, main text, or Methods section).

n/a	Confirmed	
☐	☒	The exact sample size (n) for each experimental group/condition, given as a discrete number and unit of measurement
☐	☒	An indication of whether measurements were taken from distinct samples or whether the same sample was measured repeatedly
☐	☒	The statistical test(s) used AND whether they are one- or two-sided<br>*Only common tests should be described solely by name; describe more complex techniques in the Methods section.*
☒	☐	A description of all covariates tested
☒	☐	A description of any assumptions or corrections, such as tests of normality and adjustment for multiple comparisons
☐	☒	A full description of the statistics including central tendency (e.g. means) or other basic estimates (e.g. regression coefficient) AND variation (e.g. standard deviation) or associated estimates of uncertainty (e.g. confidence intervals)
☐	☒	For null hypothesis testing, the test statistic (e.g. F, t, r) with confidence intervals, effect sizes, degrees of freedom and P value noted<br>*Give P values as exact values whenever suitable.*
☒	☐	For Bayesian analysis, information on the choice of priors and Markov chain Monte Carlo settings
☒	☐	For hierarchical and complex designs, identification of the appropriate level for tests and full reporting of outcomes
☒	☐	Estimates of effect sizes (e.g. Cohen's d, Pearson's r), indicating how they were calculated
☐	☒	Clearly defined error bars<br>*State explicitly what error bars represent (e.g. SD, SE, CI)*

Our web collection on statistics for biologists may be useful.

Software and code

Policy information about availability of computer code

Data collection	AF6000 (Leica Microsystems); BD CellQuest Pro; Evolution-Capt (Vilber Lourmat)
Data analysis	AF6000 (Leica Microsystems); FiJi (ImageJ, NIH); Microsoft Excel 2016; R; GraphPad Prism 7; BD CellQuest Pro; Bio1D (Vilber Lourmat)

For manuscripts utilizing custom algorithms or software that are central to the research but not yet described in published literature, software must be made available to editors/reviewers upon request. We strongly encourage code deposition in a community repository (e.g. GitHub). See the Nature Research guidelines for submitting code & software for further information.

Data

Policy information about availability of data

All manuscripts must include a data availability statement. This statement should provide the following information, where applicable:

- Accession codes, unique identifiers, or web links for publicly available datasets
- A list of figures that have associated raw data
- A description of any restrictions on data availability

The data that support the findings of this study are available within the paper, its supplementary information, the source data file, and from the corresponding author upon request.

April 2018

Field-specific reporting

Please select the best fit for your research. If you are not sure, read the appropriate sections before making your selection.

☒ Life sciences ☐ Behavioural & social sciences ☐ Ecological, evolutionary & environmental sciences

For a reference copy of the document with all sections, see nature.com/authors/policies/ReportingSummary-flat.pdf

Life sciences study design

All studies must disclose on these points even when the disclosure is negative.

Sample size	Independent experimental replicates: 2 or 3 (mostly 3). Number of repetitions based on standard in the field, and only as an estimate of experimental reproducibility. Cells counted for each experimental data point: 150-300, again based on standard in the field.
Data exclusions	No data were excluded for analysis.
Replication	Two or three independent experiments (performed in different days over the same strains) were performed. Results were reproducible as shown in the corresponding error bars (SE).
Randomization	microscopy fields randomly selected (near the center of the coverslip). All cells in such fields counted.
Blinding	Blinding neither possible nor relevant to this study.

Reporting for specific materials, systems and methods

Materials & experimental systems

n/a	Involved in the study	
☐	☒	Unique biological materials
☐	☒	Antibodies
☒	☐	Eukaryotic cell lines
☒	☐	Palaeontology
☒	☐	Animals and other organisms
☒	☐	Human research participants

Methods

n/a	Involved in the study	
☒	☐	ChIP-seq
☐	☒	Flow cytometry
☒	☐	MRI-based neuroimaging

Unique biological materials

Policy information about availability of materials

Obtaining unique materials	All material used in this work, including new yeast strains, is available upon request.

Antibodies

Antibodies used	1. Monoclonal Anti-c-Myc antibody produced in mouse. Sigma-Aldrich. Catalog number: M4439. Lot Number: 087M4765V. Clone: 9E10.<br>2. Monoclonal Anti-HA antibody produced in mouse. Sigma-Aldrich. Catalog number: H9658. Lot number: 075K4769. Clone HA-7.<br>3. Anti-Mouse IgG (H+L), HRP-Conjugate. Promega. Catalog number: W402B. Lot number: 0000306114. Polyclonal.
Validation	Monoclonal Anti-c-Myc and Anti-HA antibodies have been tested and used in more than 750 papers according to the manufacturer's webpage. Both are produced against human MYC oncogene and Hemagglutinin gene, respectively. We have genetically modified some of our yeast strains in order to tag the MYC and HA DNA sequences to genes of interest, being able to detect the encoded chimeric proteins by Western blot. Yeast protein tagging with these epitopes has been widely used for decades.

Flow Cytometry

Plots

Confirm that:

☐ The axis labels state the marker and fluorochrome used (e.g. CD4-FITC).

☐ The axis scales are clearly visible. Include numbers along axes only for bottom left plot of group (a 'group' is an analysis of identical markers).

☐ All plots are contour plots with outliers or pseudocolor plots.

☐ A numerical value for number of cells or percentage (with statistics) is provided.

Methodology

Sample preparation	Cells: haploid Saccharomyces cerevisiae strains (5-10 microns in size). Cells fixed and permeabilized in Ethanol 70% v/v. DNA stained with Propidium Iodide (3 microg/mL) after treatment with RNAse A and Proteinase K.
Instrument	BD FASCCalibur
Software	BD CellQuest Pro (v5.2.1)
Cell population abundance	200.000 cells analyzed after brief sonication
Gating strategy	Initial FSC/SSC settings: E00/410 (volltage) and 7.2/1.0 (AmpGain). Only a SSC threshold applied (=50). Cells positively stained with propidium iodide filtered through a threshold in the FL3 detector (=50). FL2 parameters optimized with an asyncronous culture for each experiment, so that 1C DNA content (G1 cells) lays at 20% of the FL2 linear scale, 2C (G2/M) content at 40% and 4C content (detection of endoreduplication and gain aneuploidies) at 80%. An example of the gating strategy is included in the Source Data File.

☒ Tick this box to confirm that a figure exemplifying the gating strategy is provided in the Supplementary Information.
